# Estimation and feasibility of correction modelling for mother-reported child height and weight at 2 years using data from the Australian CHAT trial

**DOI:** 10.1038/s41598-022-25388-8

**Published:** 2022-12-09

**Authors:** Yan Cheng, Huilan Xu, Chris Rissel, Philayrath Phongsavan, Limin Buchanan, Sarah Taki, Alison Hayes, Louise A. Baur, Li Ming Wen

**Affiliations:** 1 Family Planning NSW, Sydney, Australia; 2 Health Promotion Unit, Population Health Research and Evaluation Hub, Sydney Local Health District, Sydney, Australia; 3 Sydney School of Public Health, Faculty of Medicine and Health, The University of Sydney, Sydney, Australia; 4 College of Medicine and Public Health, Rural and Remote Health SA and NT, Flinders University, Darwin, Australia; 5 NHMRC Centre of Research Excellence in the Early Prevention of Obesity in Childhood (EPOCH), Sydney, Australia; 6 Specialty of Child and Adolescent Health, Sydney Medical School, The University of Sydney, Sydney, Australia

**Keywords:** Public health, Epidemiology

## Abstract

Correction modelling using reported BMI values has been employed in adolescent and adult populations to improve the accuracy of self-reporting. This study aimed to evaluate the feasibility of establishing correction modelling for mother-reported child height and weight at 2 years using data from an Australian trial in 2019. Correction modelling for BMI was conducted using mother-reported and objectively measured height and weight of 2-year-olds. Mother-reported height, weight and BMI values of 2-year-old children were adjusted based on objectively measured anthropometric data using linear regression models. ‘Direct’ and ‘indirect’ corrections were applied to the correction of BMI values. We defined the direct collection as using corrected BMI values that were predicted directly by the model and indirect correction as using corrected weight and height values to calculate corrected BMI values. Corrected BMI values via the indirect correction showed higher sensitivity or similar specificity in predicting overweight status, compared to the direct correction, and also showed higher agreement with measured values compared to the mother-reported measures. Corrected self-reported measures via an indirect correction had a better accuracy and agreement with the objectively measured data in the BMI values and classification of overweight, compared to the mother-reported values.

## Introduction

Childhood obesity affects growth and development and has become a worldwide health issue. The World Health Organization (WHO) proposed a goal of zero increase in childhood overweight by 2025^1^. In Australia, about one quarter of both children aged 2–4 years and also children and adolescents aged 2–17 years were affected by overweight or obesity in 2017–2018^2,3^. Echoing the WHO global target, Australia has developed the National Obesity Strategy 2022–2032 with the aim to reduce overweight and obesity in children and adolescents by at least 5% by 2030^3^. It is important to ensure the estimation of overweight and obesity in populations is accurate to effectively assess and monitor trends and evaluate the impact of health promotion efforts.

Body mass index (BMI; weight (kg)/height (m)^2^) is used widely to determine overweight and obesity among children^4,5^. BMI calculated by parental- or self-reported height and weight from surveys is commonly used in epidemiological studies as its feasible, efficient, and economical. However, there is a risk of both random and systematic errors embedded in the use of reported measures. For example, participants tend to overestimate their height and underestimate their weight due to social desirability, resulting in an underestimation of their actual BMI^6–9^. BMI correction, therefore, has been applied to obtain a closer approximation to the measured BMI based on the self-reported height and weight in large population studies, including in adolescents and adults^7,10–14^. However, to date few studies have used corrected BMI values in 2-year-old children. Several studies have investigated the accuracy of measured and mother-reported height and weight in children^10,15,16^ and have raised concerns about the accuracy of mother-reported anthropometry. There have been recommendations to adjust mother-reported anthropometry based on correction equations^10^.

We therefore aimed (1) to evaluate the feasibility of establishing correction modelling for mother-reported child height and weight using data from a randomised controlled trial in 2-year-old children, (2) to investigate the bias of mother-reported child anthropometric data compared with data measured objectively by researchers, and (3) to examine whether the corrected anthropometric data improved the accuracy of the mother-reported data and agreement with the objectively measured data.

## Methods

### Study context

The Communicating Healthy Beginnings Advice by Telephone Randomised Controlled Trial (CHAT) randomised controlled trial (RCT) was a three-armed RCT conducted in metropolitan Sydney, New South Wales (NSW), Australia 2017–2019. The CHAT RCT aimed to evaluate the efficacy of using telephone support or SMS, plus mailed intervention materials in promoting healthy infant feeding practices and obesity-protective behaviours in the early years of life. As part of the main outcomes, children’s height and weight were measured by research assistants (RAs) through home visiting, and also independently reported by their mother via computer assisted telephone interviewing (CATI) at 2 years of their age. The study protocol, eligibility criteria, recruitment process and outcomes are reported in detail elsewhere^17–19^. The CHAT RCT was registered with the Australian Clinical Trial Registry (ACTRN12616001470482p, 21/10/2016).

### Study participants

**Figure 1:**
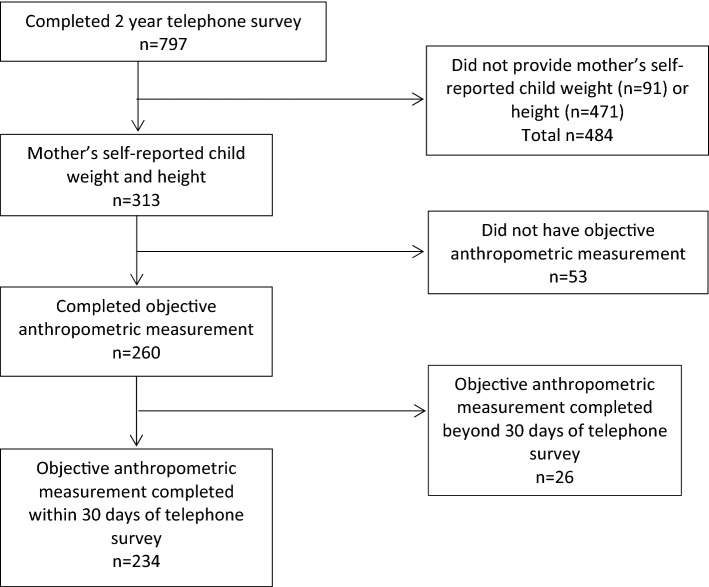
Study flowchart.

At 2 years of age, 797 (69%) completed the telephone survey^20^, of whom 484 were excluded from this analysis due to lack of mother-reported child weight or height, 53 due to the lack of anthropometric measurement and 26 due to anthropometric measurement being completed beyond 30 days of telephone survey. In total 234 participants were included in this study (Fig. 1). Participants’ demographic characteristics were compared by whether or not provision of self-reported and objectively measured anthropometric measurement at 2 years of age, showing a similar distribution in participants’ demographics between groups. However, a higher percentage of first-time mothers or mothers born overseas was observed in the group providing both self-reported and objectively measured anthropometrics (*P* < 0.05) (Supplement Table 1).

### Data collection

#### Anthropometric measurement

Anthropometric measurements were objectively measured by four RAs at the participants’ residence when the child was 2 years of age.

#### Measured height and weight

At the 2-year follow-up, an RA took height and weight measurements. Two measurements of height were made using the Seca 213 portable stadiometer and recorded it to the nearest 0.1 cm; a third measure was taken if the first two measurements differed by 0.5 cm or more and the mean of these two or three values was recorded. Weight was measured using Seca 803 digital scales on children wearing light clothes and no shoes. The measures were recorded to the nearest 0.1 kg and the mean of two measurements recorded.

#### Reported height and weight

During the same period, mother-reported height and weight were also collected within 30 days through a telephone survey. The survey was administered via telephone using CATI.

#### BMI, BMI z-score and obesity/overweight

Children’s BMI was calculated as [weight (kg)/height (m)^2^] and BMI z-score for sex and age was calculated using WHO Anthro^21^. Overweight/obesity for children at two years of age was classified based on the International Obesity Taskforce recommended age-standardised BMI cut points. A child with a BMI equals or above 18.41 for male or 18.02 for female was considered as overweight, and with a BMI equals or above 20.09 for male or 19.81 for female was considered as obesity^4^.

#### Demographics data

Sociodemographic data collected at baseline included maternal age, employment status, education level, marital status, language spoken at home, and country of birth of mothers. The questionnaires were based on the previous Healthy Beginnings Trial^22–24^.

### Statistical analysis

Descriptive analyses were undertaken to summarise the distributions of characteristics of the study participants. Considering the potential impact of the time gap between the mother-reported and the objectively measured anthropometric data on child’s height, weight and BMI outcomes, we grouped the data by the time gap, i.e., telephone-surveyed and RA-measured data within seven days or within 30 days. The distributions of participants’ characteristics were therefore described and compared by the time gap. Students t-test and Chi square test were used to test the differences between groups as appropriate. The significant level is set as two-sided *P* < 0.05, same below.

The distributions of reported and measured height, weight, calculated BMI, BMI z-score, obesity and overweight/obesity were then described for the overall sample. To identify biases in reporting, we tested for differences between reported and measured values of these outcomes using a paired t-test or McNemar’s test as appropriate. An agreement analysis was also conducted between the reported and measured data. Scatter plots were generated to show the distributions of measured height, weight and BMI calculated by measured values against reported values.

A linear regression model with measured height, weight, BMI or BMI z-score as the dependent variable and reported values of the same variable as the key independent variable was used for correction. Univariate analysis was employed to determine the selection of participants’ characteristics entering into the models, in combination with the Akaike Information Criterion. The fit of models was assessed using the adjusted-R^2^ statistic. A random normal noise term was added to the models to show the variability to the corrected values. As stated above, to evaluate the potential impact of the time gap between the reported and measured anthropometrics on the correction, two models were considered for each outcome: Model 1 for reported and measured data within 7 days (n = 111), and Model 2 for reported and measured data within 30 days (n = 234). Direct or indirect correction was applied to the correction of BMI values. Direct correction used the corrected BMI predicted by the model. Indirect correction used the corrected weight and height values to calculate corrected BMI.

We also assessed classification error using sensitivity and specificity for the binary indicators of obesity and overweight/obesity for the reported and corrected data within 7 and 30 days (Model 1–2). Sensitivity (true positive) indicates a model’s ability to correctly classify an individual with overweight or obesity while specificity (true negative) indicates its ability to correctly classify an individual with non-obesity or non-overweight/non-obesity.

Lastly, to assess the improvement of corrected estimates, we compared the agreement between corrected estimates and objectively measured data to the agreement between mother reported data and objectively measured data. Agreement analyses were undertaken and intra-class correlation coefficients (ICCs) and kappa statistics were reported to understand the extent of agreement between corrected and measured height, weight and BMI. ICC values between 0.5 and 0.75 and between 0.75 and 0.9 indicate moderate and good reliability, respectively^25^. Kappa statistics between 0.41–0.60, between 0.61–0.80 and between 0.81–1.00 suggest moderate, substantial and perfect agreement, respectively^26^. Histograms of differences between measured and self-reported height, weight and BMI values, between corrected and self-reported values, and between measured and corrected values were also generated. Data were analysed using Stata, version 13 (StataCorp LLC).

### Ethical approval and consent to participate

This study was part of the trial granted ethics approval by the Ethics Review Committee of Sydney Local Health District (Protocol No. X16–0360 & LNR/16/RPAH/495 and Protocol No X18–0387 & HREC/18/RPAH/545). Written informed consent was obtained from all study participants.

## Results

### Participants’ characteristics

The mean age of the children (47% female) at survey was 23.9 ± 0.36 months. The majority of mothers were married or had a de factor partner (96%), had a university education level (71%), were born overseas (69%), employed (67%), were first-time mothers (63%) and had a household income over $80,000 (58%). Just over half of mothers spoke English at home (51%). Two-fifths of mothers (42%) were aged 30 to 34 years, followed by 29% aged 35–39 years (Table 1).

**Table 1:** Characteristics of the study participants who were part of HB CHAT study in Sydney 2017–2019.

Demographics	Total	Time gap between telephone survey and objective measure	
	≤ 30 days n = 234	≤ 7 days n = 111	> 7 days n = 123
	n (%)	n (%)	n (%)
**Group**			
Telephone	68 (29)	32 (29)	36 (29)
SMS	91 (39)	50 (45)	41 (33)
Control	75 (32)	29 (26)	46 (37)
**Child gender**			
Male	124 (53)	62 (56)	62 (50)
Female	110 (47)	49 (44)	61 (50)
**Mother’s age, years**			
16–24	13 (6)	9 (8)	4 (3)
25–29	42 (18)	20 (18)	22 (18)
30–34	98 (42)	45 (41)	53 (43)
35–39	68 (29)	31 (28)	37 (30)
40–49	13 (6)	6 (5)	7 (6)
**Marital status**			
Married or de factor partner	224 (96)	105 (95)	119 (97)
Other	10 (4)	6 (5)	4 (3)
**Mother’s employment status**			
Employed	157 (67)	70 (63)	87 (71)
Other	77 (33)	41 (37)	36 (29)
**Annual household income**			
< $40,000	25 (11)	14 (13)	11 (9)
$40,000–$79,999	58 (25)	59 (53)	77 (63)
≥ $80,000	136 (58)	31 (28)	27 (22)
Don’t know/refused	15 (6)	7 (6)	8 (7)
**Mother’s educational level**			
Up to HSC/TAFE	68 (29)	40 (36)*	28 (23)
University	165 (71)	70 (64)	95 (77)
**Mother’s country of birth**			
Australia	72 (31)	37 (33)	35 (28)
Other	162 (69)	74 (67)	88 (72)
**Language spoken at home**			
English	120 (51)	57 (51)	63 (51)
Other	114 (49)	54 (49)	60 (49)
**First-time mother**			
Yes	148 (63)	70 (63)	78 (63)
No	86 (37)	41 (37)	45 (37)
Time gap ≤ 3 days	24 (10)		
Time gap ≤ 7 days	111 (47)		
	**Mean (SD)**	**Mean (SD)**	**Mean (SD)**
Child age at survey (months)	23.9 ± 0.36	24.0 ± 0.42	23.9 ± 0.30
Time gap between telephone survey and objective measures (days)	10.3 ± 6.86		

The sums of the variables are not the same due to missing values.

*P < 0.05.

The mean time gap between surveyed and measured anthropometrics was 10.3 ± 6.986 days, with 47% of participants having anthropometrics measured within 7 days of the telephone survey (n = 111). Similar participants’ characteristics were observed in the group with the time gap within 7 days, a lower percentage of participants with a university education (64%) was observed in the group with a time gap within 7 days (Table 1).

#### Reporting biases between reported and measured anthropometrics

On average, the reported height and weight was 0.4 cm higher and 0.1 kg lower than measured height and weight, respectively. The reported BMI and BMI z-score were both 0.2 units lower than measured BMI and BMI z-score, but the difference was not statistically significant (*P* > 0.05) (Table 2). The differences in the anthropometrics between the reported and measured anthropometrics were further analysed by different time gaps, showing the variation of differences rose as the time gap increases (Supplement Table 2). The discrepancies in measured and reported data by participants’ characteristics were described in the supplement Tables 3 and 4.

**Table 2:** Reported and objectively measured height, weight, BMI and BMI Z score in the sample.

Outcomes	Survey reported N = 234	Objective measured N = 234	Agreement analysis
	Mean (SD)	Mean (SD)	ICC (95%CI)
Height (cm)	86.5 (7.10)	86.1 (3.71)	0.42 (0.31–0.52)
Weight (kg)	12.6 (1.76)	12.7 (1.65)	0.81 (0.76–0.85)
BMI	16.7 (2.77)	16.9 (1.53)	0.28 (0.16–0.40)
BMI z-score	0.6 (1.90)	0.8 (1.10)	0.31 (0.19–0.42)
	**n (%)**	**n (%)**	**Kappa (SE)**
Obese	18 (8)	2 (1)	− 0.02(0.04)
Overweight or obese	48 (21)	49 (21)	0.26(0.07)

No significant difference was observed in the outcomes between the survey and objective measures.

The proportion of those with obesity derived from self-report was 7% higher than that derived from the measured value, but the difference was not statistically significant (*P* > 0.05). However, the proportion of those with combined overweight/obesity from the survey was the same as the measured value (Table 2).

The agreement analysis showed a good agreement (ICC = 0.81) between the reported and measured weight, a moderate agreement between the reported and measured height (ICC = 0.42), but a poor agreement between reported and measured BMI (ICC = 0.28). The Kappa coefficient of 0.26 was also low in the classification of overweight or obesity status based on reported data. The scatter plots of measured height, weight and BMI values against reported values are provided in the Supplement Figs. 1–6.

#### Quality of BMI correction models

The adjusted R^2^ statistics were higher for models with weight as a dependent variable (66–74%) compared to models of height (27–28%), BMI z-score (16–19%) and BMI (11–17%). Not surprisingly, across all outcomes (height, weight, BMI z-score and BMI), the correction models derived from reported and measured anthropometrics within 7 days (Model 1) had a consistently highest adjusted R^2^ compared to the models derived from data with a time gap within 30 days (Model 2) (Table 3). Considering the adjusted R^2^ values were all below 75%, a random normal noise with mean 0 and standard deviation equals to the square root of the mean square error from the model was added to each prediction model to show the variability to the corrected value.

**Table 3:** Quality of correction models for height, weight, BMI z-score and BMI.

Outcome^a^	Model 1: Time gap between survey and objective measures≤ 7 days^b^ (n = 111)	Model 2: Time gap between survey and objective measures≤ 30 days^c^(n = 234)
	Adjusted R^2^ %	Adjusted R^2^ %
Height(cm)	28	27
Weight(kg)	74	66
BMI z-score	19	16
BMI	17	11

^a^Selection of variables in the models is based on the univariate analysis (supplement Tables 3–4) and the Akaike Information Criterion.

^b^Included survey reported measure, age and age^2^ in the models of measured height, BMI z-score and BMI, respectively, included survey reported measure only in the model for measured weight;

^c^Include survey reported Height, age and age^2^ in the model of measured height, included survey reported weight and mother’s country of birth in the model of measured weight, included survey reported BMI z-score and household income in the model of measured BMI z-score, included survey reported BMI only in the model of measured BMI.

The correction equations for height, weight, BMI and BMI z-score are listed below.

Model 1 (time gap between the reported and measured within 7 days):

$$\begin{aligned} & Height\left( {{\text{cm}}} \right) = 11.937 + 0.307 \times Height_{MR} \left( {{\text{cm}}} \right) + 3.352 \times month - 0.057 \times month^{2} + N\left( {0, \, 3.148} \right) \\ & Weight\left( {{\text{kg}}} \right) = 2.755 + 0.783 \times Weight_{MR} \left( {{\text{kg}}} \right) \, + N\left( {0, \, 0.874} \right) \\ & BMI\,z{\text{-}}score = - {163}.{862} + 0.{257} \times BMI\,z{\text{-}}score_{MR} + {13}.{885} \times month - 0.{293} \times month^{{2}} + N\left( {0,{ 1}.0{15}} \right) \\ & BMI = - 232.846 + 0.235 \times BMI_{MR} + 20.572 \times month - 0.430 \times month^{2} + N\left( {0, \, 1.415} \right) \\ \end{aligned}$$

Model 2 (time gap between the reported and measured within 30 days):

$$\begin{aligned} & Height\left( {{\text{cm}}} \right) = - 419.150 + 0.263 \times Height_{MR} \left( {{\text{cm}}} \right) + 39.28 \times month - 0.799*month^{2} + N\left( {0, \, 3.209} \right) \\ & Weight\left( {{\text{kg}}} \right) = 3.097 + 0.765 \times Weight_{MR} \left( {{\text{kg}}} \right) - 0.282 \times mother\,born\,overseas + N\left( {0, \, 0.965} \right) \\ & BMI\, \, z{\text{-}}score = 0.264 + 0.222 \times BMI\,z{\text{-}}score_{MR} + 0.385 \times (household\,income < \$ 40,000) + 0.456 \\ & \quad \times \left( {household\,income\,between\,\$ 40,000 - \$ 79,999} \right) + 0.757 \times \left( {household\,income \ge \$ 80,000} \right) + N\left( {0, \, 1.017} \right) \\ & BMI = - 13.813 + 0.184 \times BMI_{MR} + N\left( {0, \, 1.448} \right) \\ &_{*} MR:\,mother{\text{-}}reported. \\ \end{aligned}$$

#### Accuracy of reported and corrected obesity and overweight estimates

When comparing sensitivity across reported, indirect and direct correction models using data from different time gaps (within 7 days or 30 days, respectively), indirect correction models derived from corrected anthropometrics within 7 days (Model 1) and within 30 days (Model 2) had a sensitivity of 58% and 53% for overweight/obesity respectively, and substantially higher than that of uncorrected mother’s report (around 41%). Direct correction values from Model 1 and 2 had a very low sensitivity for overweight/obesity (2–4%). The sensitivity indicator for obesity was not calculated when no child was classified as obesity, either based on the objective or corrected values. Therefore, this indicator was not available for a comparison across the models (Table 4).

**Table 4:** Sensitivity and specificity of reported and corrected outcomes.

	Model 1: Time gap between survey and objective measures≤ 7 days			Model 2: Time gap between survey and objective measures≤ 30 days		
	Survey	Indirect correction^a^	Direct correction^b^	Survey	Indirect correction^a^	Direct correction^b^
	%	%	%	%	%	%
**Sensitivity**						
Obese	N/A^c^	N/A^c^	N/A^c^	0	0	N/A^d^
Overweight or obese	41.7	58.3	4.2	40.8	53.1	2.0
**Specificity**						
Obese	N/A^c^	N/A^c^	N/A^c^	92.2	95.7	N/A^d^
Overweight or obese	85.1	86.2	97.7	84.9	85.4	96.8

^a^Indirect correction used the corrected weight and height values to calculate corrected BMI to classify overweight or obese status.

^b^Direct correction used the corrected BMI predicted by the model to classify overweight or obese status.

^c^N/A as no child was classified as obese based on objective measures.

^d^N/A as no child was classified as obese based on predicted values.

When comparing specificity across the models under ‘reported’, ‘indirect’ or ‘direct’ approaches, the specificity for overweight/obesity was highest in direct correction values (around 98%), followed by the indirect values (around 86%) and uncorrected reports (about 85%). Overall, the specificity of overweight/obesity was similar based on the indirect correction models across different time gaps (Table 4). Similar to the sensitivity indicator, the specificity indicator for obesity was not available for a comparison across the models (Table 4).

#### Agreement analysis between reported, corrected and measured outcomes

In Model 1, indirect corrected and reported means of height, weight and BMI, and overweight/obesity prevalence estimates were closer to those obtained from objective measurements. The estimate of obesity prevalence (5%) was higher than the objective value (0%). Similar to those between the reported and objective values, the ICCs between the indirect corrected and objective values ranged from 0.4 (for height) to 0.9 (for weight). However, a higher ICC for BMI (0.6) and a higher Kappa coefficient of 0.43 in the classification of overweight or obesity status were reported between the indirect corrected and objective values, compared to those between the uncorrected report or direct corrected values and objective measures (Table 5). Similar findings were observed in Model 2, where the time gap between the reported and measured data within 30 days, a higher ICC for BMI (0.6) and a higher Kappa coefficient of 0.37 in the classification of overweight or obesity status were reported between the indirect corrected and objective values, compared to those between the uncorrected report or direct corrected values and objective measures (Table 5).

**Table 5:** Intra-class correlation coefficients (ICCs) and kappa statistics between reported, corrected and measured outcomes.

Outcome	Reported		Indirect corrected^a^		Direct corrected^b^		Measured
	Mean (SD)	ICC (95%CI)	Mean (SD)	ICC (95%CI)	Mean (SD)	ICC (95%CI)	Mean (SD)
**Model 1: Time gap between reported and objective measures≤ 7 days**^**c**^** (n = 111)**							
Height (cm)	86.5 (6.40)	0.46 (0.30–0.59)	N/A	N/A	86.0 (1.95)	0.44 (0.28–0.58)	86.0 (3.67)
Weight (kg)	12.7 (1.86)	0.86 (0.80–0.90)	N/A	N/A	12.7 (1.45)	0.85 (0.79–0.89)	12.7 (1.70)
BMI	16.8 (2.54)	0.33 (0.16–0.49)	17.2 (1.68)	0.64 (0.51–0.74)	16.9 (0.64)	0.30 (0.12–0.46)	16.9 (1.53)
	**n (%)**	**Kappa (SE)**	**n (%)**	**Kappa (SE)**	**n (%)**	**Kappa (SE)**	**n (%)**
Obese	9 (8)	0 (0)	5 (5)	0 (0)	0 (0)	N/A	0 (0)
Overweight or obese	23 (21)	0.27 (0.09)	26 (23)	0.43 (0.09)	3 (3)	0.03 (0.05)	24 (22)
**Model 2: Time gap between reported and objective measures≤ 30 days**^**d**^** (n = 234)**							
Height (cm)	86.5 (7.10)	0.42(0.31–0.52)	N/A	N/A	86.1(1.90)	0.42(0.31–0.52)	86.1 (3.71)
Weight (kg)	12.6 (1.76)	0.81(0.76–0.85)	N/A	N/A	12.7(1.34)	0.80(0.74–0.84)	12.7 (1.65)
BMI	16.7 (2.77)	0.28(0.16–0.40)	17.1(1.63)	0.56(0.47–0.65)	16.9(0.51)	0.20(0.07–0.32)	16.9 (1.53)
	**n (%)**	**Kappa (SE)**	**n (%)**	**Kappa (SE)**	**n (%)**	**Kappa (SE)**	**n (%)**
Obese	18 (8)	− 0.02(0.04)	10(4)	− 0.01(0.05)	0(0)	N/A	2 (1)
Overweight or obese	48 (21)	0.26(0.07)	53(23)	0.37(0.07)	7(3)	− 0.02(0.04)	49 (21)

^a^Indirect correction used the corrected weight and Height values to calculate corrected BMI.

^b^Direct correction used the corrected weight, height and BMI predicted by the model.

^c^Only included reported and measured anthropometrics within 7 days.

^d^Only included reported and measured anthropometrics within 30 days.

Histograms of differences between measured and self-reported height, weight and BMI values, between corrected and self-reported values, and between measured and corrected values were shown in Supplement Figs. 7–28, separated by different time gaps. The Figures show a decreased difference when the time gap between the reported and measured data was within 7 days, and between the measured and corrected values when compared to the differences between the measured and self-reported values (Supplement Figs. 7–28).

## Discussion

### Principal findings of the study

Findings from our study have shown a poor agreement in BMI and classification of overweight between the mother-reported and objectively measured data. We therefore aimed to explore the feasibility of establishing correction modelling for mother-reported child height and weight at 2 years of age. The correction modelling has shown an improvement in the accuracy and agreement in BMI values and the classification of overweight compared to the mother-reported data. Further, we have found the correction modelling in height, weight, BMI z-score and BMI reported a better fit if the time gap between the occurrence of survey and objective measurement was within seven days (Model 1), compared to that within 30 days (Model 2). Also, corrected BMI values via an indirect correction (i.e., use of the corrected weight and height values to calculate corrected BMI) showed a higher sensitivity and similar specificity, and a better agreement with measured values compared to the corrected BMI values derived from a direct correction (i.e., use of corrected BMI predicted by the model in both Models 1 and 2).

### Meaning of the study

Although correction modelling of BMI has been evaluated in the population of adolescents and adults^7,10–14^, we are not aware of a similar study that has been reported in children at 2 years of age. Our analyses have shown a poor agreement between mother-reported and objectively-measured data, consistent with previous studies in children^10,15,16^, suggesting the necessity of correction modelling based on mother-reported data in children. The corrected BMI values from the correction modelling have shown a better accuracy and agreement with the measured data, compared to the mother-reported data. Our findings suggest corrected anthropometric data (i.e., BMI) from mothers reported value can be considered as an option to allow health professionals to monitor children growth and development.

### What the study adds

Our findings suggest corrected BMI value had a better accuracy and agreement with the objectively measured BMI value compared to reported data, especially when the survey and measurement occurred within a short timeframe that is within seven days. These findings might be attributed to the child’s growth, or be impacted by the chance of increasing the magnitude of errors as the sample size of participants increases, as shown in the supplement data, where the variation of difference between reported and measured anthropometrics increased as more data were included. Further study is warranted in terms of the impact of timeframe to collect reported and measured data on correction modelling.

Our findings also showed BMI values corrected via an indirect correction (i.e., calculated by corrected height and weight) had a better performance than the direct correction (i.e., corrected BMI values predicted by the model) in terms of accuracy in predicting overweight/obese status and agreement with the objectively measured BMI value. The better performance of indirect correction model was consistent with a previous study in adolescents^11^, which suggested undertaking BMI correction using the corrected height and weight values to calculate corrected BMI, rather than taking the direct corrected BMI values predicted by the BMI correction model. This could be explained by the better fit of models (higher R^2^) in the height and weight correction models than the direct BMI correction model.

Across the correction models, weight correction models consistently showed a better fit when compared to the correction models in the height, BMI z-score and BMI. The variation of difference in weight between measured and reported data was lower than the height. Feedback from the RAs also reflected the difficulty in measuring the height over weight among children in this study. Therefore, a guideline in measurement of children’s height and weight should be developed to support parents in collecting more acute anthropometric data.

### Unanswered questions and future research

Apart from the correction modelling of weight, the correction models of height and BMI had a lower fit than a previous correction modelling study in adolescents^11^. Further study to explore the feasibility of correction modelling in a large sample of children is warranted.

Although misreporting was found to be related to gender, ethnicity and other socioeconomic status in other studies^10,15,16^, we did not find many discrepancies in this study. The role of socioeconomic-status bias in the BMI correction modelling study in children needs to be further investigated.

### Strengths and limitations

To our knowledge, this is the first study to investigate the feasibility of correction modelling of BMI based on mother-reported height and weight in children of 2 years age. However, our study has several limitations. First, the sample size was relatively smaller than in other correction modelling studies^7,11,12^. The differences between the mother-reported and objectively measured data were not significant partially due to the lack of statistical power resulting from the small sample size. It is important to replicate our results for less common outcomes such as obesity status with larger sample sizes. Second, due to the small sample size, we were not able to assess the model’s predictive capacity on a separate dataset that was not used to develop this model. Finally, the majority of mothers included in the correction modelling study were from a culturally and linguistically diverse background, with a university education level and were first-time mother, all these may contribute to a less reporting bias of child’s height and weight, therefore limit the generalisability of our findings.

## Conclusion

In this study we found a poor agreement between reported and measured data. Corrected anthropometric data via an indirect correction has a better accuracy and agreement with the objectively measured data in terms of BMI values and classification of overweight, compared to the mother-reported values. The correction modelling had a better fit if the time gap between measured and reported data within seven days. Our findings suggest corrected anthropometric data (e.g., BMI) from mothers-reports can be considered as an option to allow health professionals to monitor children growth and development.

## Supplementary Information

Supplementary Information.

## Data Availability

The datasets used and/or analysed during the current study will be available from the corresponding author upon reasonable request and ethics approval after the completion of the study in June 2023.
