# Supplementary material for: Estimation and feasibility of correction modelling for mother-reported child height and weight at 2 years using data from the Australian CHAT trial
Source: Sci Rep. 2022 Dec 9;12:21353. doi: 10.1038/s41598-022-25388-8 (PMC9734091; doi:10.1038/s41598-022-25388-8)
Supplement: Supplementary file 1 — Supplementary Information. [file 41598_2022_25388_MOESM1_ESM.docx]

**Appendix: supplemental tables and figures**

Table 1 Participants’ demographic characteristics by provision of mother’s self-reported and objectively measured anthropometric measurement at 2 years of age.

| **Baseline demographics** | **Total** | **Provision of mother’s self-reported and objectively measured anthropometric measurement at 2 years** | |
| --- | --- | --- | --- |
|  | **N=797 (%)** | **Yes**  **n=260 (%)** | **No**  **n=537 (%)** |
| **Child gender** |  |  |  |
| Male | 413 (52) | 125 (52) | 278 (48) |
| Female | 384 (48) | 135 (48) | 259 (52) |
| **Mother’s age** |  |  |  |
| 16-24 | 52 (6) | 15 (6) | 37 (7) |
| 25-29 | 172 (22) | 44 (17) | 128 (24) |
| 30-34 | 318 (40) | 111 (43) | 207 (39) |
| 35-39 | 200 (25) | 74 (28) | 126 (23) |
| 40-49 | 55 (7) | 16 (6) | 39 (7) |
| **Mother’s country of birth*** |  |  |  |
| Australia | 299 (37) | 76 (29) | 223 (42) |
| Other | 498 (63) | 184 (71) | 314 (58) |
| **Mother’s language spoken at home** |  |  |  |
| English | 426 (53) | 134 (52) | 292 (54) |
| Other | 371 (47) | 126 (48) | 245 (46) |
| **Annual household income** |  |  |  |
| < $ 40,000 | 82 (10) | 27 (10) | 55 (10) |
| $ 40,000 to $79,999 | 171 (22) | 62 (24) | 109 (20) |
| ≥ $ 80,000 | 466 (58) | 150 (58) | 316 (59) |
| Don’t know/Refused | 78 (10) | 21 (8) | 57 (11) |
| **Mother’s employment status 2 groups** |  |  |  |
| Employed  (employed/paid/unpaid maternity leave) | 521 (65) | 174 (67) | 347 (65) |
| Other | 276 (35) | 86 (33) | 190 (35) |
| **Mother’s marital status** |  |  |  |
| Married/de-facto partner | 754 (95) | 248 (95) | 506 (94) |
| Other | 42 (5) | 12 (5) | 30 (6) |
| **Mother’s education level** |  |  |  |
| Up to HSC to TAFE/Diploma | 564 (71) | 184 (71) | 380 (71) |
| University | 232 (29) | 75 (29) | 157 (29) |
| **First time mother*** |  |  |  |
| Yes | 436 (55) | 167 (64) | 269 (50) |
| No | 361 (45) | 93 (36) | 268 (50) |

Note: total number is not always 797, 260 and 537 due to missing values

*: P value of Chi square test <0.05

Table 2 Difference between measured and reported height, weight, BMI z-score and BMI by time gap between telephone survey and objective measures

| Outcome | Time gap between reported and objective measures | |  |
| --- | --- | --- | --- |
|  | **<=7 days**  **n=111** | **<=30 days**  **n=234** | |
| Difference in height |  |  | |
| Mean (SD) | -0.514 (5.441) | -0.416 (6.119) | |
| Min | -14.0 | -16.25 | |
| Max | 23.0 | 33.78 | |
| Range | 37.0 | 50.03 | |
| Difference in weight |  |  | |
| Mean (SD) | 0.003 (0.959) | 0.046 (1.060) | |
| Min | -4.35 | -5.70 | |
| Max | 3.10 | 5.10 | |
| Range | 7.45 | 10.80 | |
| Difference in BMI z-score |  |  | |
| Mean (SD) | 0.143 (1.653) | 0.193 (1.811) | |
| Min | -6.29 | -7.29 | |
| Max | 4.80 | 5.01 | |
| Range | 11.09 | 12.30 | |
| Difference in BMI |  |  | |
| Mean (SD) | 0.085 (2.431) | 0.168 (2.683) | |
| Min | -11.40 | -12.60 | |
| Max | 5.20 | 8.00 | |
| Range | 16.60 | 20.60 | |

Note: all differences reported here are measured values minus reported values.

Table 3 Difference between reported and measured height, weight, BMI z-score and BMI by demographic groups (time gap between reported and measured outcomes less than 7 days) (n=111)

| Demographics | | Difference between reported and measured height | | Difference between reported and measured weight | | Difference between reported and measured BMI z-score | | Difference between reported and measured BMI | |
| --- | --- | --- | --- | --- | --- | --- | --- | --- | --- |
|  |  | Mean | SD | Mean | SD | Mean | SD | Mean | SD |
| Group | Telephone | 1.2 | 6.21 | 0.0 | 0.92 | -0.3 | 2.04 | -0.7 | 3.25 |
|  | SMS | -1.6 | 4.48 | 0.0 | 0.99 | 0.5 | 1.21 | 0.7 | 1.69 |
|  | Control | -0.6 | 5.73 | -0.1 | 0.97 | 0.1 | 1.75 | 0.0 | 2.27 |
| Child gender | Male | -0.4 | 4.60 | 0.0 | 0.93 | 0.1 | 1.55 | 0.1 | 2.25 |
|  | Female | -0.6 | 6.40 | 0.0 | 1.01 | 0.1 | 1.79 | 0.1 | 2.67 |
| Mother’s age, y | 16-24 | -4.1 | 7.13 | -0.4 | 1.59 | 0.8 | 2.10 | 1.0 | 2.56 |
|  | 25-29 | 0.7 | 2.81 | 0.2 | 0.92 | 0.0 | 1.03 | -0.1 | 1.44 |
|  | 30-34 | -0.9 | 6.68 | 0.0 | 0.78 | 0.2 | 1.88 | 0.2 | 2.77 |
|  | 35-39 | -0.2 | 3.54 | 0.0 | 0.79 | 0.0 | 1.41 | -0.1 | 2.25 |
|  | 40-49 | 1.8 | 5.93 | 0.5 | 1.77 | -0.1 | 2.20 | -0.5 | 3.42 |
| Mother’s country of birth | Australia | -0.3 | 5.49 | 0.0 | 1.30 | 0.1 | 1.66 | -0.1 | 2.58 |
|  | other | -0.6 | 5.45 | 0.0 | 0.75 | 0.2 | 1.66 | 0.2 | 2.37 |
| Language spoken at home | English | -0.5 | 4.89 | 0.0 | 1.16 | 0.2 | 1.50 | 0.1 | 2.30 |
|  | other | -0.5 | 6.02 | 0.0 | 0.70 | 0.1 | 1.81 | 0.0 | 2.58 |
| Annual household income | <$ 40,000 | 0.0 | 7.49 | -0.3 | 1.33 | -0.2 | 1.90 | -0.3 | 2.60 |
|  | >=$ 80,000 | -0.3 | 5.48 | 0.0 | 0.92 | 0.1 | 1.67 | 0.0 | 2.68 |
|  | $ 40,000 to $79,999 | -0.5 | 3.90 | 0.0 | 0.91 | 0.2 | 1.26 | 0.2 | 1.76 |
|  | Don't know/refused | -3.0 | 6.83 | 0.4 | 0.50 | 1.3 | 2.29 | 1.3 | 2.50 |
| Mother’s employment status | Employed | -0.1 | 5.31 | 0.1 | 0.93 | 0.0 | 1.64 | -0.1 | 2.58 |
|  | Other | -1.3 | 5.63 | -0.1 | 1.01 | 0.3 | 1.69 | 0.3 | 2.15 |
| Marital status | Married/de-facto partner | -0.3 | 5.25 | 0.0 | 0.87 | 0.1 | 1.64 | 0.0 | 2.43 |
|  | Other | -3.9 | 7.98 | -0.4 | 2.03 | 0.9 | 1.94 | 1.2 | 2.36 |
| Mother’s educational level | Up to HSC/TAFE | -0.5 | 5.46 | 0.0 | 1.14 | 0.1 | 1.57 | 0.1 | 2.04 |
|  | University | -0.5 | 5.50 | 0.0 | 0.85 | 0.1 | 1.72 | 0.1 | 2.65 |
| First-time mother | Yes | -0.5 | 6.01 | 0.0 | 0.97 | 0.2 | 1.75 | 0.1 | 2.51 |
|  | No | -0.5 | 4.36 | 0.0 | 0.95 | 0.1 | 1.49 | 0.0 | 2.31 |

Note: no significant differences were observed in reported and measured outcomes across demographic groups.

Table 4 Difference between reported and measured height, weight, BMI z-score and BMI by demographic groups (time gap between reported and measured outcomes less than 30 days) (n=234)

| Demographics | | Difference between reported and measured height | | Difference between reported and measured weight | | Difference between reported and measured BMI z-score | | Difference between reported and measured BMI | |
| --- | --- | --- | --- | --- | --- | --- | --- | --- | --- |
|  |  | Mean | SD | Mean | SD | Mean | SD | Mean | SD |
| Group | Telephone | 0.4 | 6.44 | 0.1 | 1.04 | -0.1 | 2.09 | -0.3 | 3.26 |
|  | SMS | -0.9 | 5.33 | 0.1 | 0.96 | 0.4 | 1.66 | 0.4 | 2.42 |
|  | Control | -0.6 | 6.69 | -0.1 | 1.19 | 0.2 | 1.72 | 0.2 | 2.37 |
| Child gender | Male | -0.3 | 6.02 | 0.0 | 1.17 | 0.2 | 1.80 | 0.1 | 2.65 |
|  | Female | -0.5 | 6.25 | 0.1 | 0.92 | 0.2 | 1.83 | 0.2 | 2.73 |
| Mother’s age, y | 16-24 | -1.4 | 8.69 | -0.2 | 1.35 | 0.2 | 2.40 | 0.1 | 3.25 |
|  | 25-29 | 1.8 | 6.94 | 0.1 | 1.26 | -0.2 | 1.81 | -0.4 | 2.93 |
|  | 30-34 | -1.2 | 6.12 | 0.1 | 0.79 | 0.4 | 1.77 | 0.5 | 2.53 |
|  | 35-39 | -0.5 | 4.89 | -0.1 | 1.17 | 0.1 | 1.74 | 0.0 | 2.62 |
|  | 40-49 | -0.5 | 5.20 | 0.3 | 1.31 | 0.4 | 1.80 | 0.4 | 2.75 |
| Mother’s country of birth | Australia | -0.2 | 5.87 | -0.2* | 1.37 | -0.2* | 1.92 | -0.4* | 2.99 |
|  | other | -0.5 | 6.24 | 0.2 | 0.87 | 0.4 | 1.74 | 0.4 | 2.51 |
| Language spoken at home | English | -0.7 | 5.15 | 0.0 | 1.23 | 0.2 | 1.77 | 0.2 | 2.69 |
|  | other | -0.2 | 7.01 | 0.1 | 0.84 | 0.2 | 1.86 | 0.2 | 2.69 |
| Annual household income | <$ 40,000 | 0.3 | 6.15 | -0.3 | 1.35 | -0.4* | 1.50 | -0.5* | 2.03 |
|  | >=$ 80,000 | -0.6 | 5.99 | 0.1 | 0.99 | 0.2 | 1.89 | 0.2 | 2.91 |
|  | $ 40,000 to $79,999 | 0.7 | 6.16 | 0.1 | 1.14 | 0.0 | 1.61 | 0.0 | 2.42 |
|  | Don't know/refused | -3.9 | 6.20 | 0.3 | 0.67 | 1.4 | 1.87 | 1.5 | 2.02 |
| Mother’s employment status | Employed | -0.1 | 6.26 | 0.1 | 1.06 | 0.2 | 1.87 | 0.2 | 2.85 |
|  | Other | -1.1 | 5.80 | -0.1 | 1.06 | 0.2 | 1.71 | 0.2 | 2.31 |
| Marital status | Married/de-facto partner | -0.3 | 6.11 | 0.1 | 1.03 | 0.2 | 1.82 | 0.1 | 2.71 |
|  | Other | -2.7 | 6.18 | -0.2 | 1.62 | 0.6 | 1.52 | 0.9 | 1.87 |
| Mother’s educational level | Up to HSC/TAFE | 0.2 | 6.97 | -0.1 | 1.06 | 0.0 | 1.61 | -0.1 | 2.21 |
|  | University | -0.7 | 5.75 | 0.1 | 1.06 | 0.3 | 1.89 | 0.3 | 2.86 |
| First-time mother | Yes | -0.3 | 6.60 | 0.1 | 0.97 | 0.2 | 1.87 | 0.3 | 2.77 |
|  | No | -0.5 | 5.21 | -0.1 | 1.20 | 0.1 | 1.71 | 0.0 | 2.53 |

*: P<0.05 comparing the difference between reported and measured outcomes by mother’s country of birth and household income.

Figure S1 Scatter plot of measured against self-reported height for time gap <=7 days (cm)


Figure S2 Scatter plot of measured against self-reported height for time gap <=30 days (cm)

Figure S3 Scatter plot of measured against self-reported weight for time gap <=7 days (kg)

Figure S4 Scatter plot of measured against self-reported weight for time gap <=30 days (kg)

Figure S5 Scatter plot of BMI calculated by measured against self-reported weight for time gap <=7 days

Figure S6 Scatter plot of BMI calculated by measured against self-reported weight for time gap <=30 days

Figure S7 Histogram of difference between measured and self-reported height (cm) for time gap <=7 days

Figure S8 Histogram of difference between measured and self-reported height (cm) for time gap <=30 days

Figure S9 Histogram of difference between corrected and self-reported height (cm) for time gap <=7 days

Figure S10 Histogram of difference between corrected and self-reported height (cm) for time gap <=30 days

Figure S11 Histogram of difference between measured and corrected height (cm) for time gap <=7 days

Figure S12 Histogram of difference between measured and corrected height (cm) for time gap <=30 days

Figure S13 Histogram of difference between measured and self-reported weight (kg) for time gap <=7 days

Figure S14 Histogram of difference between measured and self-reported weight (kg) for time gap <=30 days

Figure S15 Histogram of difference between corrected and self-reported weight (kg) for time gap <=7 days

Figure S16 Histogram of difference between corrected and self-reported weight (kg) for time gap <=30 days

Figure S17 Histogram of difference between measured and corrected weight (kg) for time gap <=7 days

Figure S18 Histogram of difference between measured and corrected weight (kg) for time gap <=30 days

Figure S19 Histogram of difference between BMI calculated by measured and self-reported value for time gap <=7 days

Figure S20 Histogram of difference between BMI calculated by measured and self-reported value for time gap <=30 days

Figure S21 Histogram of difference between BMI calculated by direct corrected and self-reported value for time gap <=7 days

Figure S22 Histogram of difference between BMI calculated by direct corrected and self-reported value for time gap <=30 days

Figure S23 Histogram of difference between BMI calculated by indirect corrected and self-reported value for time gap <=7 days

Figure S24 Histogram of difference between BMI calculated by indirect corrected and self-reported value for time gap <=30 days

Figure S25 Histogram of difference between BMI calculated by measured and direct corrected value for time gap <=7 days

Figure S26 Histogram of difference between BMI calculated by measured and direct corrected value for time gap <=30 days

Figure S27 Histogram of difference between BMI calculated by measured and indirect corrected value for time gap <=7 days

Figure S28 Histogram of difference between BMI calculated by measured and indirect corrected value for time gap <=30 days
